# Supplementary material for: In Vitro Bioaccessibility and Antioxidant Activity of Coffee Silverskin Polyphenolic Extract and Characterization of Bioactive Compounds Using UHPLC-Q-Orbitrap HRMS
Source: Molecules. 2020 May 2;25(9):2132. doi: 10.3390/molecules25092132 (PMC7249082; doi:10.3390/molecules25092132)
Supplement: Supplementary file 1 [file molecules-25-02132-s001.pdf]

**Supplementary materials:**

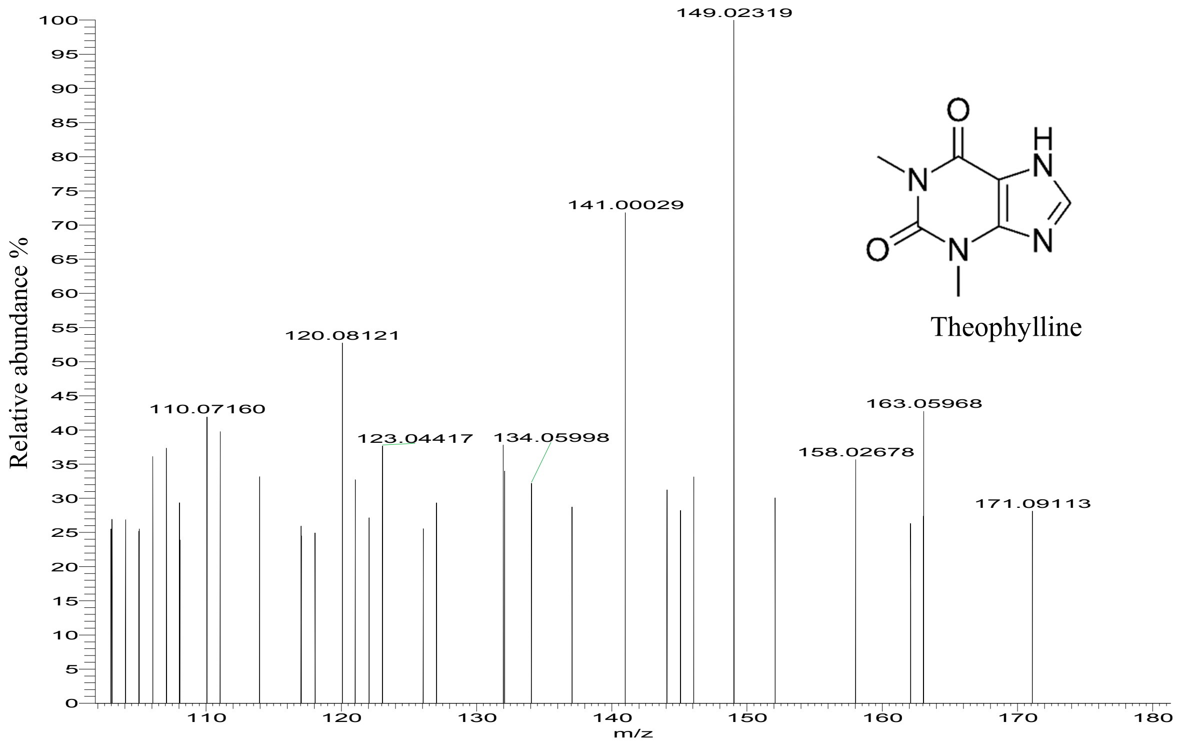


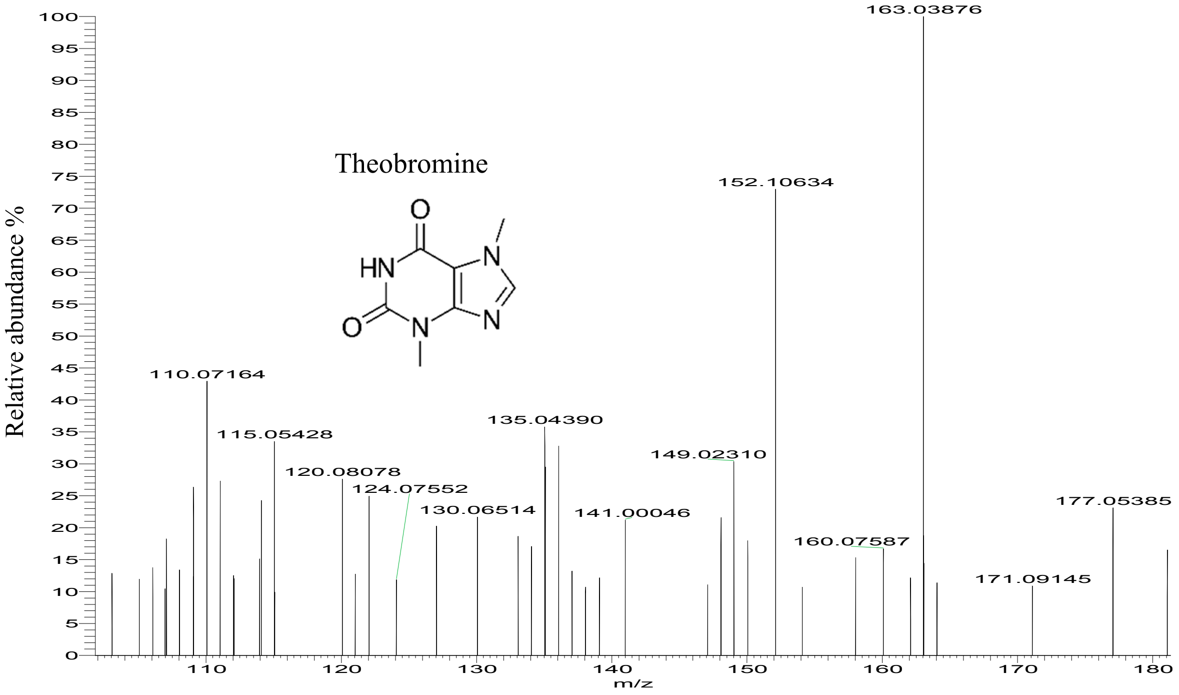

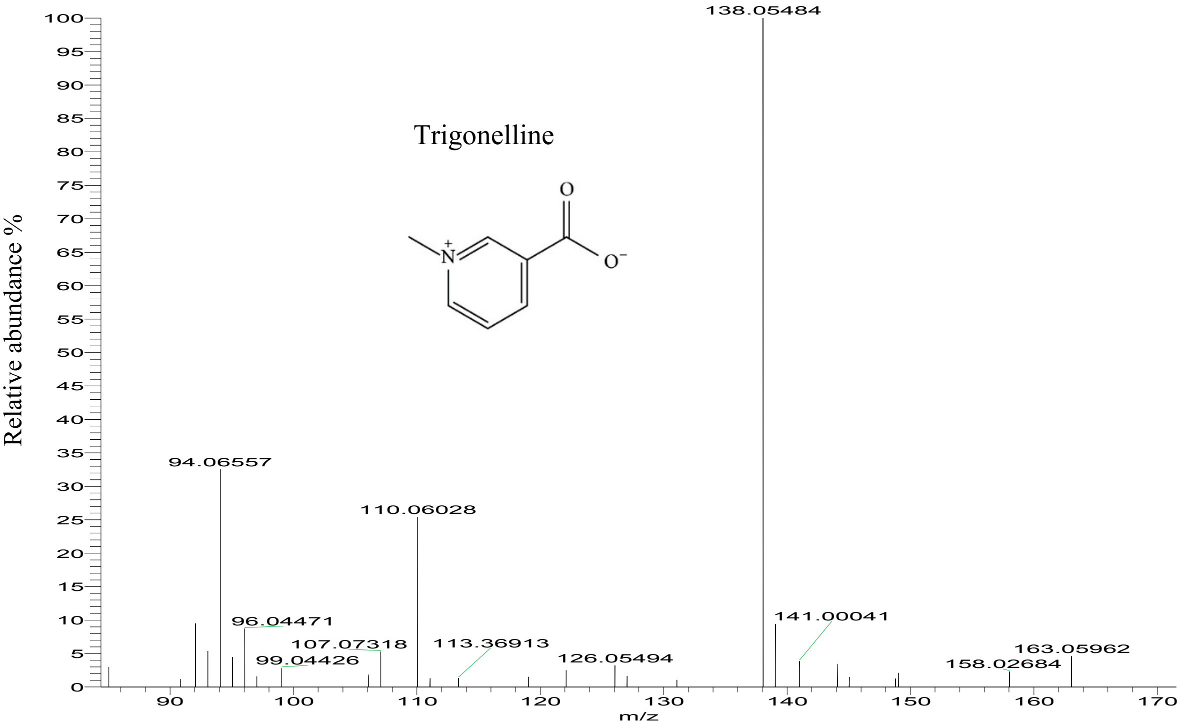

Trigonelline
138.05484
94.06557
110.06028
96.04471
107.07318
99.04426
113.36913
126.05494
141.00041
163.05962
158.02684
Relative abundance %
m/z


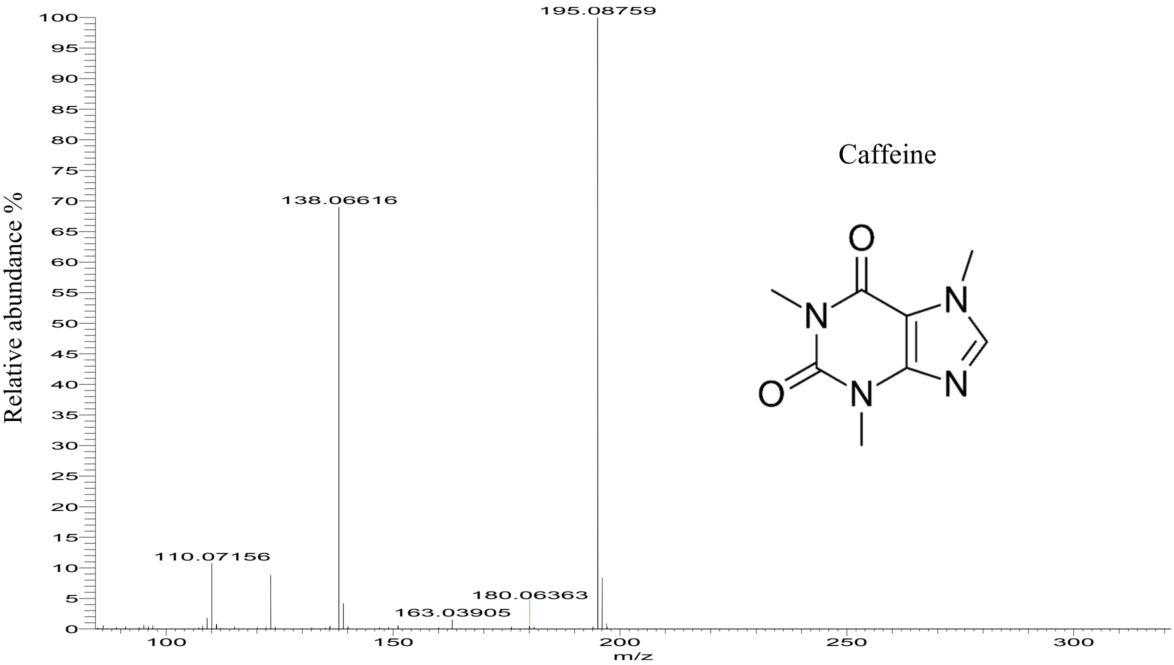

Caffeine
195.08759
138.06616
110.07156
180.06363
163.03905
Relative abundance %
m/z

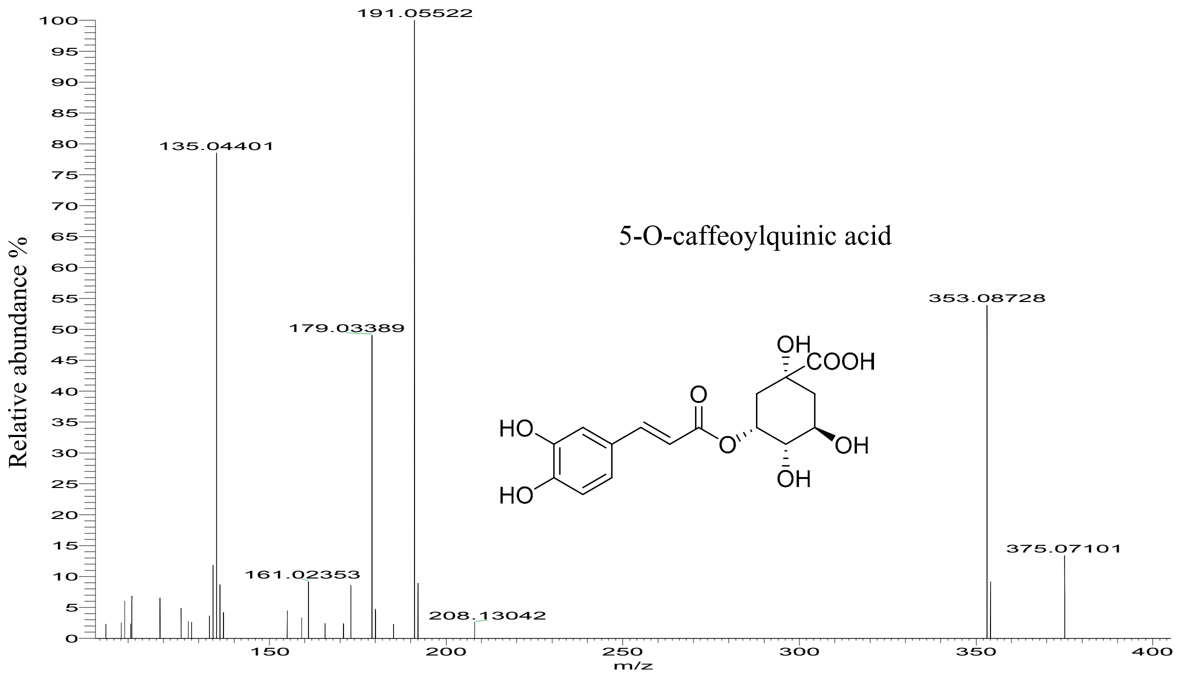
5-O-caffeoylquinic acid
Relative abundance %
m/z
191.05522
135.04401
179.03389
161.02353
208.13042
353.08728
375.07101

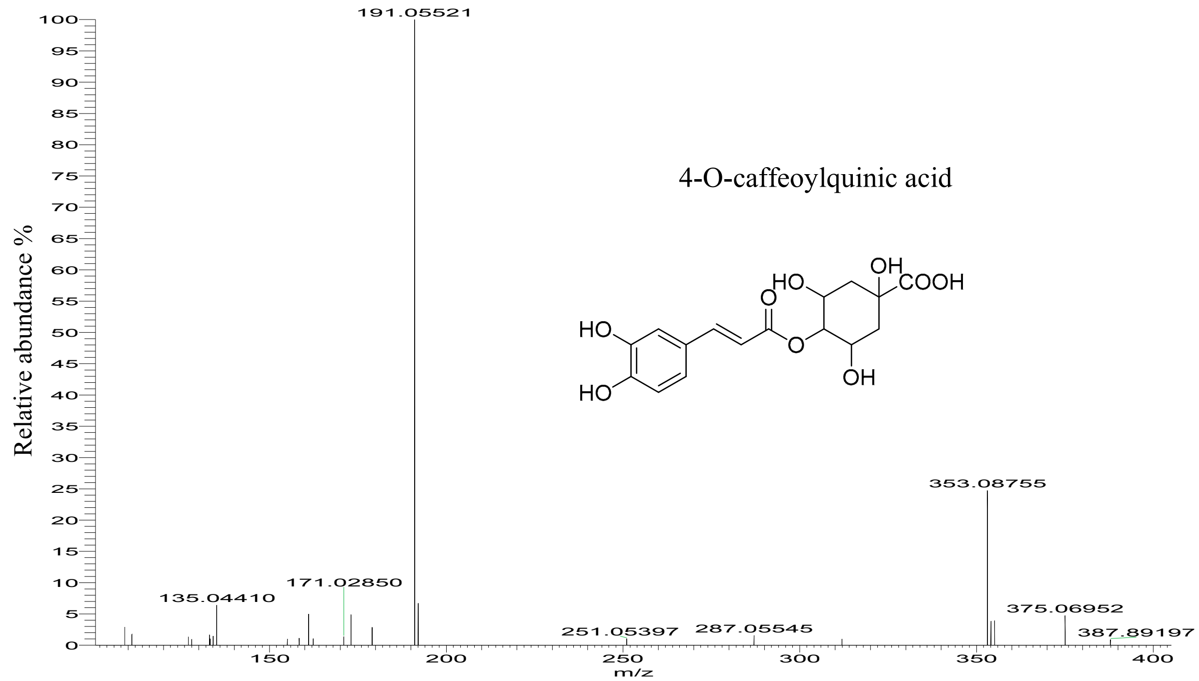
4-O-caffeoylquinic acid
Relative abundance %
m/z
191.05521
135.04410
171.02850
251.05397
287.05545
353.08755
375.06952
387.89197

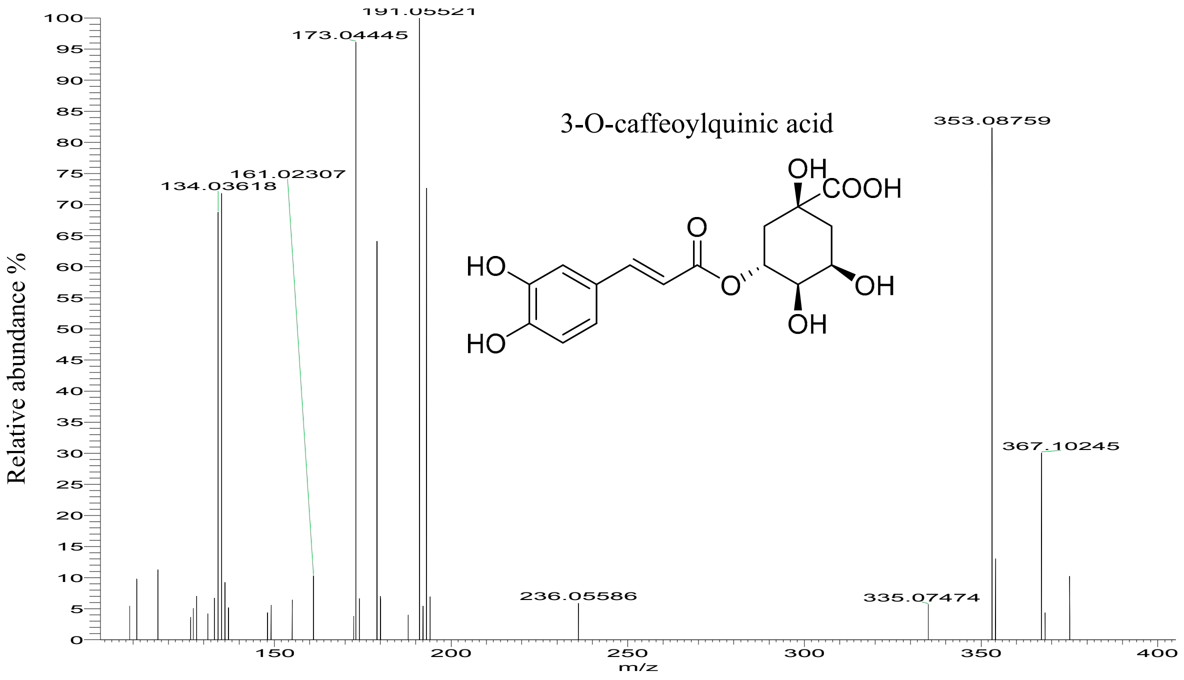

191.05521
173.04445
3-O-caffeoylquinic acid
353.08759
161.02307
134.03618
OH
COOH
O
HO
O
OH
OH
HO
367.10245
236.05586
335.07474
Relative abundance %
m/z


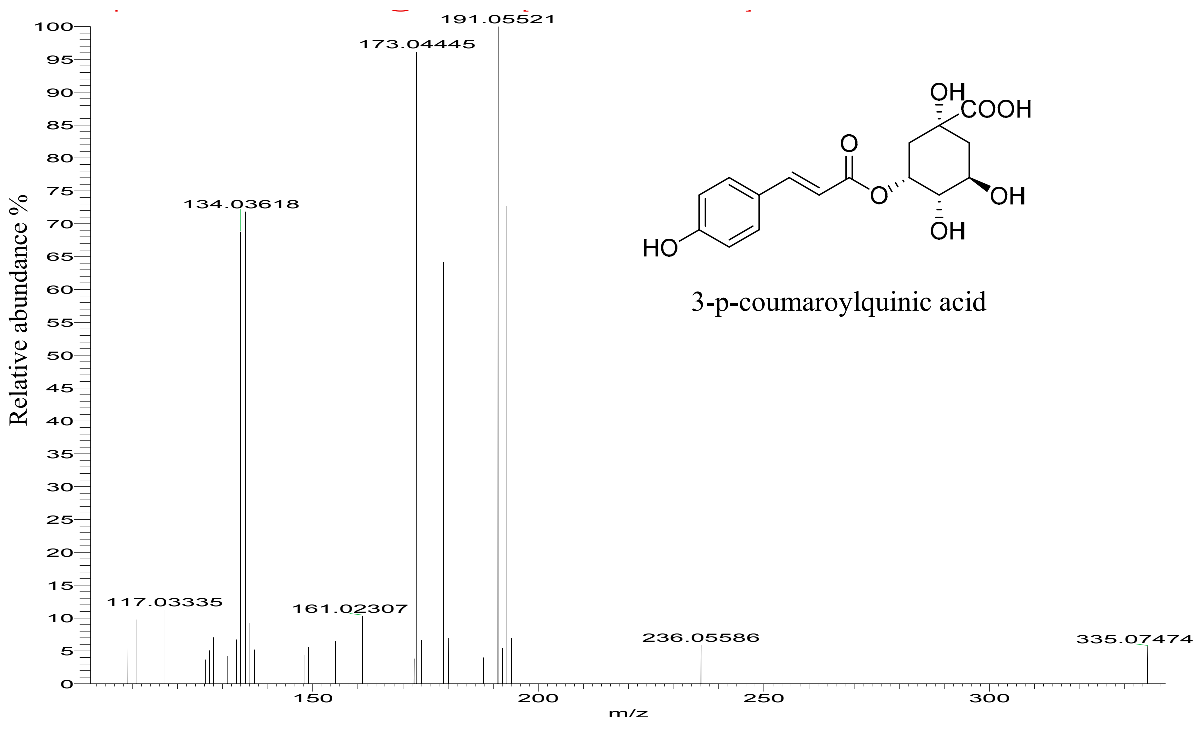

191.05521
173.04445
OH
COOH
O
134.03618
O
OH
HO
OH
3-p-coumaroylquinic acid
117.03335
161.02307
236.05586
335.07474
Relative abundance %
m/z

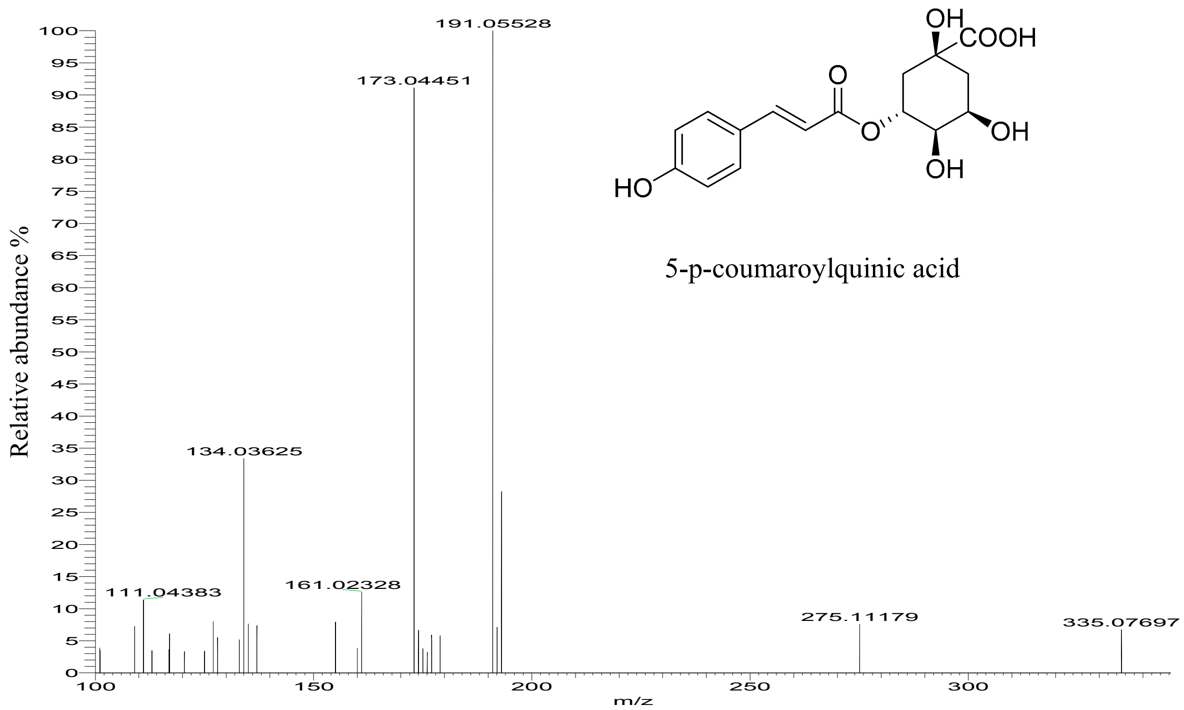
Relative abundance %
m/z
191.05528
173.04451
134.03625
111.04383
161.02328
275.11179
335.07697
5-p-coumaroylquinic acid

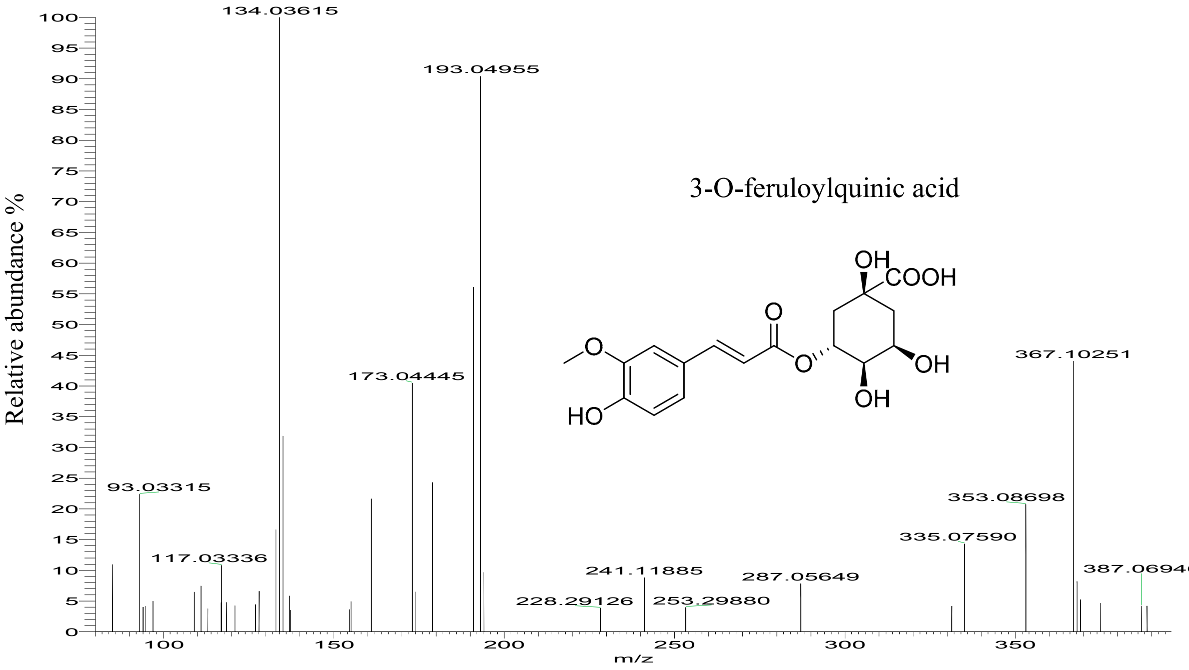
Relative abundance %
m/z
134.03615
193.04955
173.04445
93.03315
117.03336
228.29126
241.11885
253.29880
287.05649
335.07590
353.08698
367.10251
387.06946
3-O-feruloylquinic acid

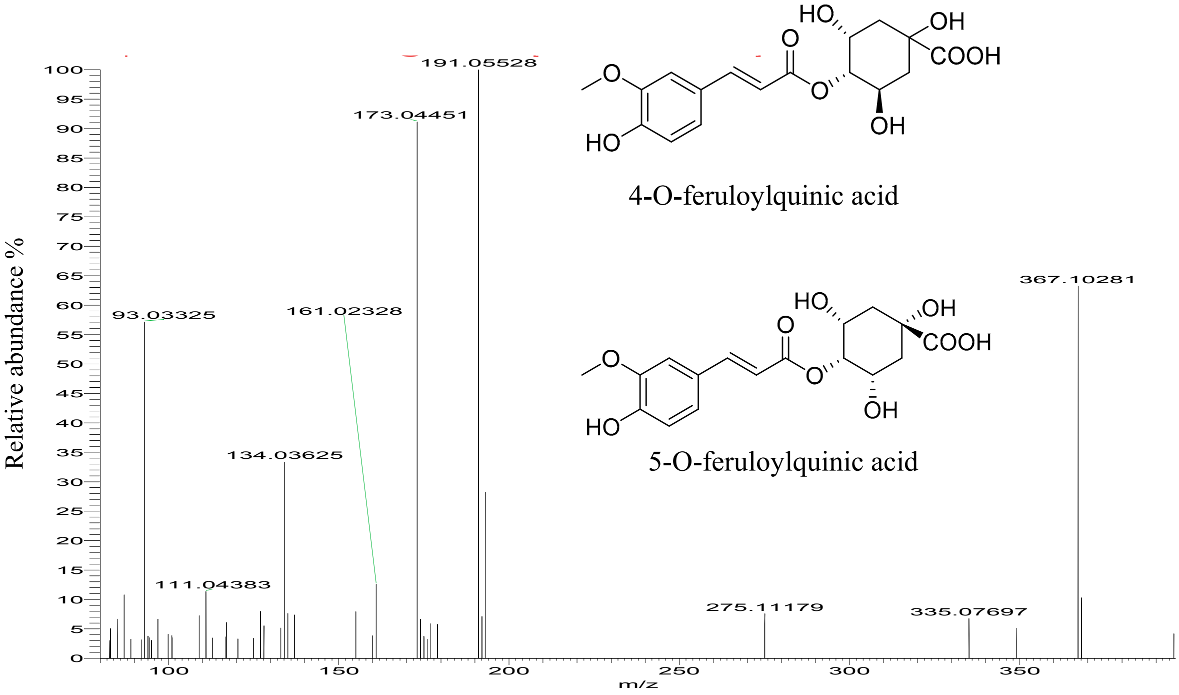

4-O-feruloylquinic acid
5-O-feruloylquinic acid
Relative abundance %
m/z
191.05528
173.04451
367.10281
161.02328
93.03325
134.03625
111.04383
275.11179
335.07697


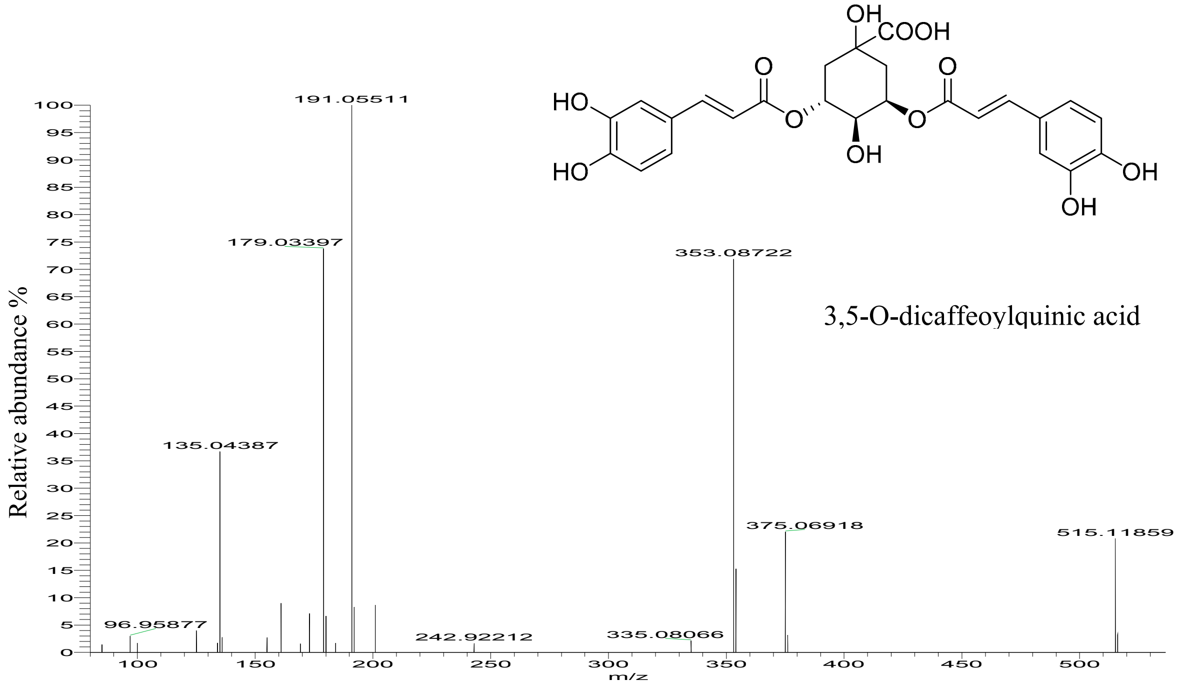

3,5-O-dicaffeoylquinic acid
Relative abundance %
m/z
191.05511
179.03397
353.08722
135.04387
375.06918
515.11859
96.95877
242.92212
335.08066

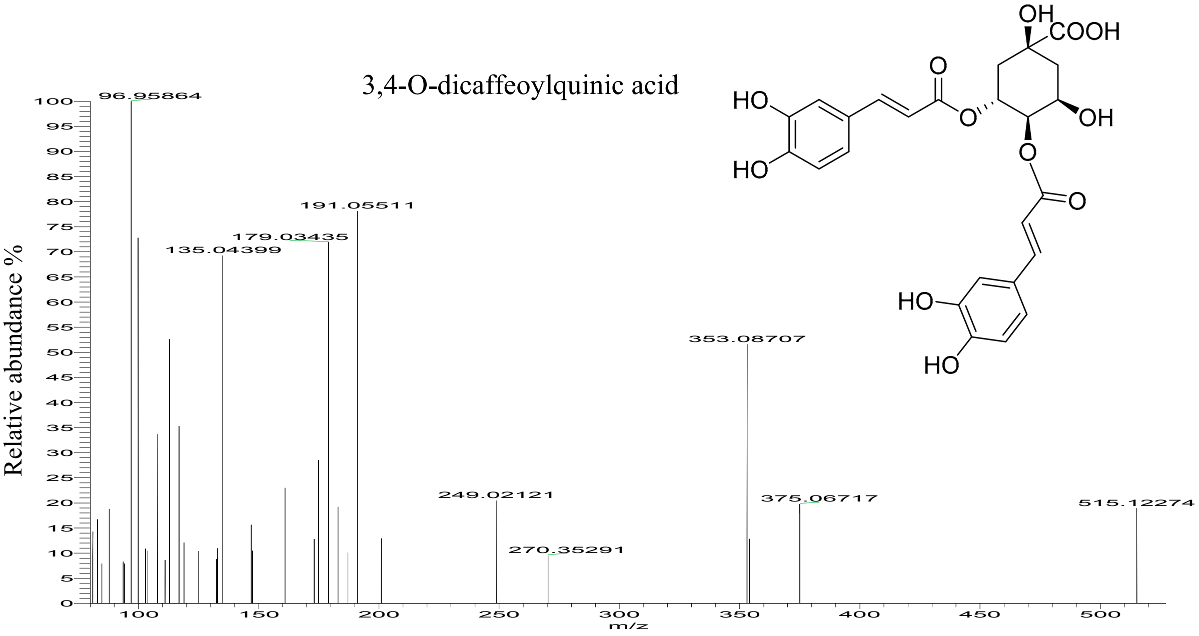
3,4-O-dicaffeoylquinic acid
Relative abundance %
m/z
96.95864
191.05511
179.03435
135.04399
353.08707
249.02121
375.06717
515.12274
270.35291
OH
COOH
HO
HO
O
OH

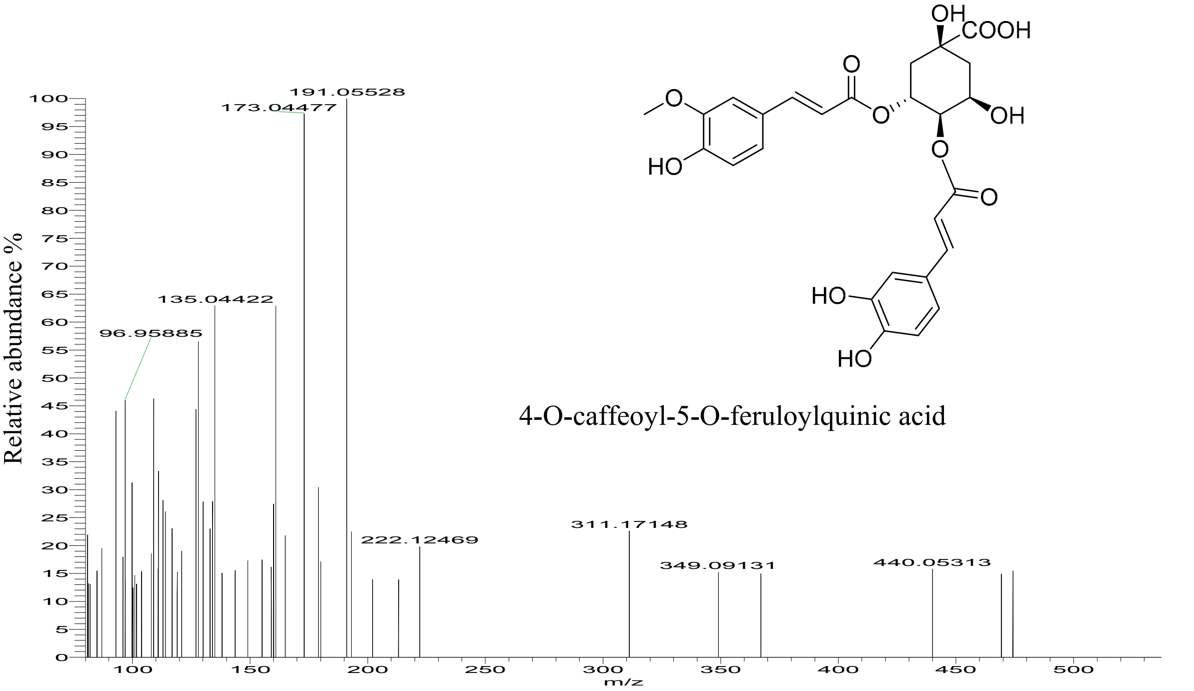
4-O-caffeoyl-5-O-feruloylquinic acid
Relative abundance %
m/z
191.05528
173.04477
135.04422
96.95885
222.12469
311.17148
349.09131
440.05313
OH
COOH
HO
O
OH

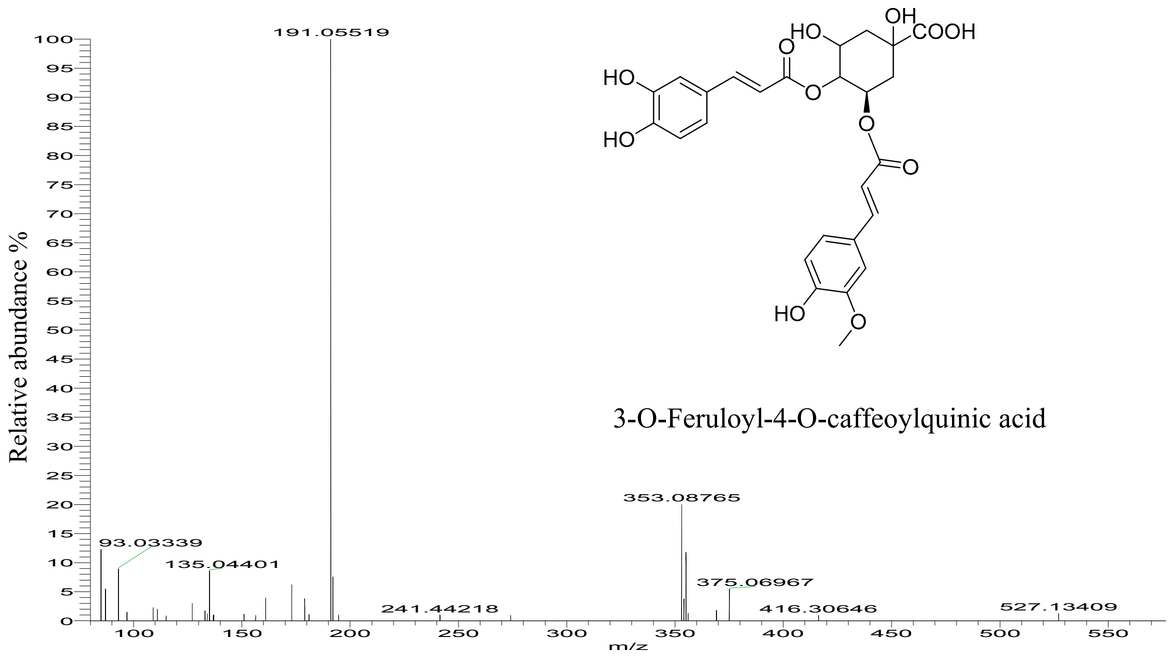


**Supplementary Figure S1.** MS/MS mass spectra of investigated compounds ($n$ = 15) extracted of coffee silverskin produced by roasting coffee beans (*Coffea arabica* L.).
